# Diagnostic and treatment implications of psychosis secondary to treatable metabolic disorders in adults: a systematic review

**DOI:** 10.1186/1750-1172-9-65

**Published:** 2014-04-28

**Authors:** Olivier Bonnot, Hans Hermann Klünemann, Frederic Sedel, Sylvie Tordjman, David Cohen, Mark Walterfang

**Affiliations:** 1 Department of Child and Adolescent Psychiatry, Centre Hospitalier Universitaire de Nantes, Hôpital Mère-Enfant, 7 quai Moncousu, 44 000 Nantes, France; 2 Klinik und Poliklinik für Psychiatrie, Psychosomatik und Psychotherapie der Universität Regensburg am Bezirksklinikum, Regensburg, Germany; 3 Department of Neurology and Reference Center for Lysosomal Diseases, Groupe Hospitalier Pitié-Salpêtrière, Paris, France; 4 Department of Child and Adolescent Psychiatry, Centre Hospitalier Guillaume Regnier, University of Rennes, Rennes, France; 5 Department of Child and Adolescent Psychiatry and Reference Center for Rare Diseases with Psychiatric Symptoms, Groupe Hospitalier Pitié-Salpêtrière, Paris, France; 6 Neuropsychiatry Unit, Royal Melbourne Hospital, Melbourne, Australia & Melbourne Neuropsychiatry Centre, University of Melbourne, Melbourne, Australia; 7 GDR3557-Institut de Psychiatrie, Paris, France

**Keywords:** Inborn errors of metabolism, Organic psychosis, Schizophrenia-like symptoms, Atypical psychosis

## Abstract

**Objective:**

It is important for psychiatrists to be aware of certain inborn errors of metabolism (IEMs) as these rare disorders can present as psychosis, and because definitive treatments may be available for treating the underlying metabolic cause. A systematic review was conducted to examine IEMs that often present with schizophrenia-like symptoms.

**Data sources:**

Published literature on MEDLINE was assessed regarding diseases of homocysteine metabolism (DHM; cystathionine beta-synthase deficiency [CbS-D] and homocysteinemia due to methyltetrahydrofolate reductase deficiency [MTHFR-D]), urea cycle disorders (UCD), acute porphyria (POR), Wilson disease (WD), cerebrotendinous-xanthomatosis (CTX) and Niemann-Pick disease type C (NP-C).

**Study selection:**

Case reports, case series or reviews with original data regarding psychiatric manifestations and cognitive impairment published between January 1967 and June 2012 were included based on a standardized four-step selection process.

**Data extraction:**

All selected articles were evaluated for descriptions of psychiatric signs (type, severity, natural history and treatment) in addition to key disease features.

**Results:**

A total of 611 records were identified. Information from CbS-D (n = 2), MTHFR-D (n = 3), UCD (n = 8), POR (n = 12), WD (n = 11), CTX (n = 14) and NP-C publications (n = 9) were evaluated. Six non-systematic literature review publications were also included. In general, published reports did not provide explicit descriptions of psychiatric symptoms. The literature search findings are presented with a didactic perspective, showing key features for each disease and psychiatric signs that should trigger psychiatrists to suspect that psychotic symptoms may be secondary to an IEM.

**Conclusion:**

IEMs with a psychiatric presentation and a lack of, or sub-clinical, neurological signs are rare, but should be considered in patients with atypical psychiatric symptoms.

## Introduction

A range of medical conditions may be associated with schizophrenia-like psychosis [1]. The landmark review of psychosis associated with organic disorders by Davison and Bagley, which utilized the 1957 WHO operational criteria for schizophrenia, highlighted a number of disorders where the association with psychosis significantly exceeded chance [2]. Many of the disorders identified showed pathology in the temporal lobe and diencephalon.

A large study of 268 consecutive patients with first-episode psychosis found that 6% had organic cerebral disease that was potentially causally linked with psychiatric symptoms [3], which emphasizes the importance of a thorough diagnostic evaluation to exclude underlying medical illness at first presentation. Recently, more than 60 different congenital conditions associated with psychosis were reviewed. Interestingly, some of them are not associated with dysmorphia, mental retardation or prominent neurological features that may otherwise trigger a search for an organic cause of illness [4].

The diagnosis of medical or neurological illnesses underlying psychosis is of great importance as many of these conditions are progressive or fatal, associated with significant additional medical comorbidity, and may be partially or entirely reversible with definitive treatment. Inborn errors of metabolism (IEMs) represent a particular focus for research as they are frequently under-detected or misdiagnosed, a number are treatable, and new diagnostic methods and therapies have become available.

IEMs are a group of diseases that generally result from the absence or deficiency of an intracellular component of a metabolic pathway (usually, but not exclusively, an enzyme), which may lead to altered intracellular synthesis and catabolism [5]. There are hundreds of IEMs, and many remain poorly characterized. Most result in clinical disease due to the accumulation of substances that are toxic to, or interfere with, normal cellular function, or which may be due to the effects of a reduced ability to synthesize essential compounds.

The overall incidence of IEMs has been estimated to be approximately 40 cases per 100 000 live births [6]. However, this rate may be an underestimation, as new disorders continue to be discovered and characterized and because diagnostic techniques continue to improve in sensitivity and accuracy.

Up to 80% of IEMs are diagnosed during childhood, but an increasing recognition of late-onset presentations has recently raised awareness and diagnoses of adult-onset forms [7]. A number of adult-onset IEMs are associated with schizophrenia-like symptoms, including diseases of homocysteine metabolism (DHM), urea cycle disorders (UCD), porphyria (POR), Wilson disease (WD), cerebrotendinous xanthomatosis (CTX) and Niemann-Pick disease type C (NP-C) [7].

This article reports findings from a systematic literature review and provides a guide for the diagnosis of treatable IEMs associated with schizophrenia-like symptoms based on the review findings. The major features of IEMs that can be associated with psychosis are summarized, and a diagnostic algorithm to assist psychiatrists in the detection of atypical symptoms is proposed that may be related to underlying IEMs.

## Methods

### Review scope

A meeting was held in mid-2012 to decide which IEMs associated with psychosis are currently treatable, with the aim of conducting a systematic bibliographic search to address the clinical challenges associated with these conditions. Based on a consensus reached during that meeting, the following seven IEMs were chosen as a focus for this review: homocysteinemia due to methyltetrahydrofolate reductase deficiency (MTHFR-D), cystathionine beta-synthase deficiency (CbS-D), UCD, POR, WD, CTX and NP-C.

### Literature search methodology and data sources

**Figure 1:**
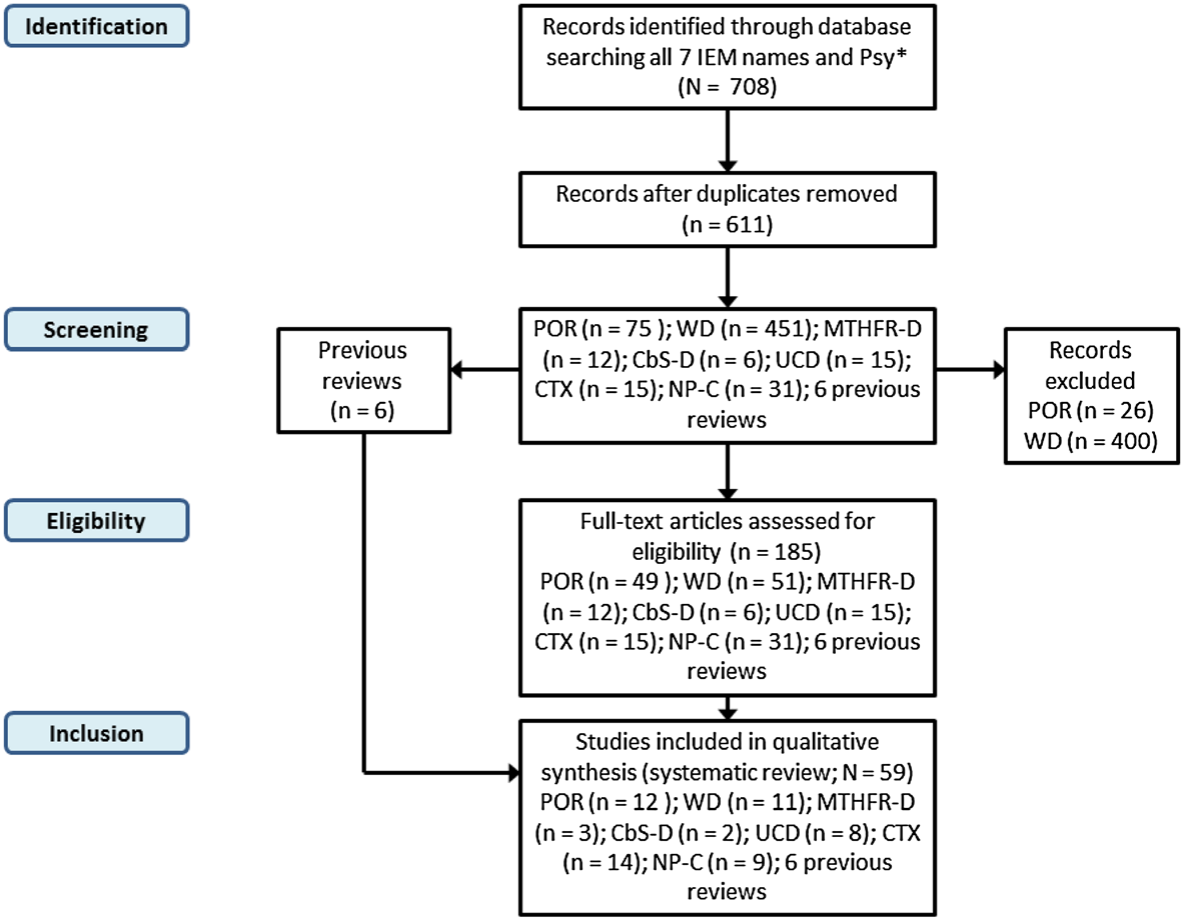
Summary of literature review process.

The public MEDLINE database was searched according to a standard four-step protocol, as described in the following sections and summarized in Figure 1.

#### Identification

All terms, including complete names and abbreviations for MTHFR-D, CbS-D, UCD, POR, WD, CTX and NP-C were searched alongside the generic tag, ‘psy*’ using EndNote X5 software (Thomson Reuters), which enabled the identification and deletion of any duplicates. In total, 708 potentially relevant records published between January 1967 and June 2012 were identified, from which 97 duplicate records were removed. Seven separate EndNote databases were created – one for each IEM. The numbers of articles for each IEM database were: MTHFR-D (n = 12); CbS-D (n = 6); UCD (n = 15); POR (n = 75); WD (n = 451); CTX (n = 15); and NP-C (n = 31). Six non-systematic literature reviews were also identified and included [7-12]. A total of 611 records were collated for screening.

#### Screening

Two groups worked separately in screening abstracts from relevant articles from the literature review (Group 1: M. Walterfang and H.H. Kluenemann. Group 2: O. Bonnot, D. Cohen, Sylvie Tordjman and F. Sedel). Case reports, case series with original data regarding psychiatric manifestations and cognitive impairments, and previous reviews containing relevant data were selected. Articles were excluded from full text analysis (see ‘*Eligibility’* stage) according to the following exclusion criteria: 1) the article mentioned psychiatric manifestations without data pertaining to any of the seven chosen treatable diseases; 2) an unrelated article, mentioning an IEM without describing psychiatric presentations; 3) literature reviews not containing any new data and; 4) data already reported elsewhere. Screening excluded 26 of the initial records from the POR database and 400 records from the WD database. In cases where the two analysis groups did not agree, records were kept and included in the next step.

#### Eligibility

The same two analysis groups accessed the full texts of all remaining articles (n = 185) and checked them further for eligibility according to the same exclusion criteria used in the abstract screening stage. The numbers of articles considered eligible after this process were: MTHFR-D (n = 12), CbS-D (n = 6), UCD (n = 15), POR (n = 49), WD (n = 51), CTX (n = 15) and NP-C (n = 31). The six previous reviews were also kept.

#### Inclusion

Among the eligible records, information on key IEM disease features as well as psychiatric manifestations was included from the following numbers of publications, per database: MTHFR-D (n = 3); CbS-D (n = 2); UCD (n = 8); POR (n = 12); WD (n = 11); CTX (n = 14) and; NP-C (n = 9). Including the six previous reviews, this brought the final total of source articles to 59.

## Results

To understand the metabolic pathways implicated in these disorders, we will provide an explanation in the text referring directly to the cited diseases. For figures and complete presentation, we refer the reader to the KFGG website which illustrates theses pathways in detail [13].

### Disorders of homocysteine metabolism (DHMs)

CbS-D and MTHFR-D are two key DHMs that commonly feature psychiatric signs.

#### Key features of CbS-D

Homocysteinuria due to CbS-D is characterized by the involvement of the ocular, skeletal, central nervous and vascular systems. Prevalence is estimated around 1/344 000 birth in countries were systematic search of CbS *deficiency* is provided for every newborn, however recent data from systematic search for CbS *mutation* show important prevalence up to 1/20 000 [14]. Two articles that addressed psychiatric symptoms and psychosis were identified [15,16]. The disease is an autosomal recessive disorder of methionine metabolism, caused by mutations in the *CbS* gene (21q22.3). CbS normally converts homocysteine to cystathionine in the trans-sulfuration pathway of the methionine cycle, and requires pyridoxal 5-phosphate as a cofactor. The other two cofactors involved in methionine remethylation include vitamin B_12_ and folic acid. Clinical diagnosis of CbS-D is confirmed by blood amino acid analysis (including total homocysteine measurement), assays of CbS enzyme activity, or screening for *CbS* mutations.

Patients appear normal at birth but display a progressive disease course if left untreated. Eye anomalies include ectopia lentis (in 85% of cases) and high myopia. Skeletal changes include genu valgum and pes cavus, followed by dolichostenomelia, pectus excavatum or carinatum, kyphoscoliosis and osteoporosis. A Marfan-like body habitus may occur, with tall stature and arachnodactyly. Thromboembolism affecting both large and small arteries and veins is the most striking cause of morbidity and mortality, and affects 25% of individuals by the age of 15 years. While some individuals have a normal IQ, mental retardation is common and, when present, may progress if the disorder is left untreated. Brittle hair and livedo reticularis have also been reported.

#### Psychiatric signs associated with CbS-D

In one of the few studies in this field, psychiatric illness was found in 51% of cases overall, with symptoms falling into four diagnostic categories: episodic depression (10%), chronic disorders of behavior (17%), obsessive-compulsive disorder (5%), and personality disorder (19%) [15]. In the same study, aggressive behavior and other conduct disorders were particularly common among patients with mental retardation and those who were nonresponsive to vitamin B_6_. In some cases, psychiatric symptoms may be the initial presenting symptom with no neurological signs [16].

#### Key features of MTHFR-D

MTHFR-D is another autonomic recessive trait, and is caused by mutations in the 5,10-methylenetetrahydrofolate reductase (*MTHFR*) gene (1p36.3). MTHFR deficiency results in abnormal intracellular folic acid metabolism, and prevents reduction of 5–10 methylenetetrahydrofolate to 5-methyltetrahydrofolate – the methyl donor for the remethylation of homocysteine into methionine. As a result, the disorder leads to MTHFR-D and consequently to homocysteinuria and hypomethioninemia. To our knowledge prevalence is unknown.

The onset of MTHFR-D usually occurs during the first year of life, characterized by severe neurological signs, recurrent apnea, microcephaly and convulsions without megaloblastic anemia. However, there are some forms with onset during childhood, adolescence or adulthood that present with mental regression, ataxia and schizophrenia-like psychosis. Other symptoms such as sub-acute degeneration of the spinal cord have been reported.

Diagnoses of DHM are made through analysis of amino acids by chromatography and total plasma homocysteine measurement; an elevated level is defined at >100 micromol/L [17]. Methionine levels may be useful, as they are decreased in MTHFR-D and increased in CbS-D.

There are currently three recognized treatment modalities for DHM. For pyridoxine-responsive patients, treatment with pharmacological doses of pyridoxine combined with folic acid and vitamin B_12_ supplements is recommended. In pyridoxine non-responsive patients, the treatment should comprise a methionine-restricted, cysteine-supplemented diet in combination with the pyridoxine, folic acid and vitamin B_12_ supplementation. Betaine anhydrous is a methyl donor that may lead to lowering of homocysteine levels in MTHFR-D patients, and can be used as an adjunct to such a diet.

Homocysteine is cleared by transulfuration to cysteine and glutathione, an important antioxidant. Transulfuration requires vitamins B_6_ and B_12_. Treatment with vitamin B_6_, a precursor of homocysteine, can be effective in treating psychiatric symptoms if instituted early [7].

#### Psychiatric signs associated with MTHFR-D

Three articles were identified from the systematic literature review [18-20]. Psychiatric symptoms are not uncommon in MTHFR-D, and may be the presenting symptom [18]. Their onset may be acute or may follow a more insidious course. Acute manifestations occur mainly after surgery, and present with visual and/or auditory hallucinations, thought disorder and delusions [18]. Roze and colleagues describe two siblings (16- and 24-year-old women) with presumed late-onset MTHFR-D. Three years after the diagnosis of her older sister, the 16-year-old sister was initially seen with a 3-month history of dissociative symptoms and delusions of persecution with visual and auditory hallucinations. One month prior to hospital admission, she had also developed an unsteady gait and urinary incontinence. She was previously healthy and had been an average student. Physical examination on admission showed only an areflexic paraparesis with an extensor plantar response on the right side and impaired vibration and position sense in the lower limbs. Diagnosis was made because her sister was known to have the disease. Combined daily treatment with intravenous hydroxocobalamin (2 mg), oral betaine (9 g), L-carnitine (3 g), and folinic acid (10 mg) was started, and a dramatic clinical improvement, with the recovery of arm function and the disappearance of psychotic features and lethargy, was observed after 6 weeks.

In a different approach, a recent meta-analysis examining the association between *MTHFR* gene polymorphisms and psychiatric disorders demonstrated a strong association between the *MTHFR* C677T gene variant and unipolar depression, schizophrenia and bipolar disorder, with odds ratios of 1.36, 1.44 and 1.82, respectively [20]. Notably, the metabolic syndrome secondary to antipsychotic medication may be more frequent in patients with reduced MTHFR activity associated with schizophrenia-like psychosis [21].

It is well documented that aberrant methylated compounds are linked to mental state and behavior. A recent review of the ‘one-carbon metabolism hypothesis’ described a range of factors that can contribute to folate and/or vitamin B_12_ deficiency [22]. Moreover, folic acid is a water-soluble B vitamin involved in the synthesis, repair and methylation of DNA, leading to epigenetic regulation of crucial developmental genes implicated in the pathogenesis of schizophrenia [23,24]. Deficiency of B vitamins leads to an increased level of homocysteine, which is a highly toxic metabolite to neural and vascular development [25]. Elevated serum levels of homocysteine have also been shown to be associated with schizophrenia, although the evidence is far from conclusive [26,27].

### Urea cycle disorders (UCDs)

#### Key features

The urea cycle is the metabolic process by which the body eliminates nitrogen. Six enzymes take part in this process; a deficiency of any one of them disrupts this pathway and results in excess nitrogen accumulating in the body in the form of ammonia. The six UCDs include deficiency of: 1) carbamyl phosphate synthetase; 2) n-acetylglutamate synthetase; 3) ornithine transcarbamylase; 4) argininosuccinic acid synthetase (also called citrullinemia); 5) argininosuccinase acid lyase and; 6) arginase. UCD has an estimated incidence of 1:8.000 [28].

If the enzyme deficiency is severe, symptoms will be present at birth and can present as irritability, nausea and vomiting followed by lethargy, seizures and poor muscle tone. If left untreated, patients can develop respiratory distress or fall victim to coma or premature death due to pathological levels of ammonia in the blood.

If the enzyme deficiency is partial, symptom onset may not occur until childhood or adulthood. In such cases, symptoms may include nausea and vomiting associated with headache and a clouded sensorium in the context of infection or a high-protein diet. Medications may worsen or trigger the disease, particularly corticosteroids and sodium valproate.

There is no cure for UCDs, although prompt diagnosis allows measures to be taken that can reduce the consequences of hyperammonemia. Measurement of plasma ammonemia is key to the diagnosis of UCDs, and treatment consisting of a protein-restricted diet and special supplements is essential [29]. In addition, several medications including sodium benzoate, sodium phenylacetate and sodium phenylbutyrate can bind with ammonia and remove it from the circulation. Hemodialysis may represent an alternative treatment, especially in emergency situations [30].

#### Psychiatric signs

Eight relevant articles were identified in the literature review [31-38]. Arn and colleagues [31] reported a 21-year-old white woman who presented 8 days postpartum with headache and confusion, and became uncommunicative. She was admitted to a psychiatric hospital and diagnosed with postpartum depression. Within 24 hours of admission (11 days postpartum) she became comatose and had generalized tonic–clonic seizures, decorticate posturing, and papilledema. She was treated successfully with hemodialysis and received intravenous sodium benzoate and arginine hydrochloride. Enns and colleagues reported a similar case, suggesting that UCD may present initially with postpartum psychiatric symptoms and may represent an under-recognized cause of ‘postpartum psychosis.’ [32]

Interestingly, late-onset (between 13 and 48 years of age in our review) UCD may present with behavioral and hallucinatory psychiatric and organic signs, often featuring vomiting, which is clearly a key trigger sign for the consideration of UCD in psychiatric situations [35-37]. Patients may have a history of anorexia and atypical depression [35] or psychosis [36], often with associated confusion [37].

### Acute porphyria (POR)

#### Key features

The PORs comprise a group of eight hereditary metabolic diseases characterized by intermittent neurovisceral manifestations, cutaneous lesions, or the combination of both. All porphyrias are caused by a deficiency in one of the enzymes of the heme biosynthesis pathway. These deficiencies result in an accumulation of porphyrins and/or their precursors – delta-aminolevulinic acid (ALA) and porphobilinogen (PBG) – in the liver or bone marrow. Neurological manifestations are caused by the neurotoxic effects of these precursors, particularly ALA. Prevalence is estimated around 0.54:100 000 [39].

Enzyme deficiencies in the porphyrias result from mutations of the correspondingly coded genes, and transmission of hereditary porphyrias occurs in either an autosomal dominant fashion with weak penetrance, or in a recessive manner with complete penetrance.

Clinical signs of disease usually appear in adulthood, although some porphyrias affect children. Porphyrias are classified into two groups – hepatic and erythropoietic – according to the main location of the metabolic anomaly. Chronic hepatic porphyrias and erythropoietic porphyrias manifest with bullous cutaneous lesions or acute pain in areas exposed to the sun, without neurological symptoms. However, neuro-visceral attacks occur in patients with acute hepatic porphyrias, manifesting as intense abdominal pain (often associated with nausea, vomiting and constipation), and neurological and psychological symptoms. Two acute hepatic porphyrias (variegate porphyria and hereditary coproporphyria) may also present with cutaneous photosensitivity.

Diagnosis is mainly based on the measurement of porphyrins and their precursors in biological samples such as urine, stools and blood. The key diagnostic procedure is the measurement of ALA and PBG in urine. Genetic counseling should be offered to affected families to identify individuals susceptible to developing and transmitting the disease. Acute attacks should be treated urgently with an injection of human hemin and/or perfusion of carbohydrates.

#### Psychiatric signs

Twelve relevant articles were included from the systematic literature review. Psychiatric manifestations are widely known and well documented [40-43], occurring in 24–70% of patients in acute porphyria series [44,45]. The most common manifestations reported are delirium, psychosis and depression, with some authors suggesting that 40% of acute porphyrias present with delirium and hallucinations [46].

Some cases are spectacular. For example, Santosh and Malhotra reported the case of a 13- year-old boy with six episodes of psychosis with various presentations, including delusions, hallucinations, hypomania and catatonia, but with no obvious organic signs [43]. Crimlisk described three noteworthy cases. The first was that of a 53-year-old woman with a history of cognitive decline since the age of 20, and episodes of visual hallucinations, ataxia, abdominal pain and weight loss; diagnosis was achieved after an acute vomiting episode at 53 years of age. A 52-year-old woman with a history of episodic psychiatric disturbance with associated ideas of reference, auditory hallucinations, emotional lability and abdominal pain was also described, with episodes tending to occur pre-menstrually. The third case was a young boy with a history of generalized pain, vesicular rash, fever and nausea, in whom a diagnosis was made when psychiatric symptoms (paranoid schizophrenia) appeared.

Finally, Mandoki and Summer reported a sub-acute psychiatric dysphoric presentation with emotional lability and aggression, as well as headaches and abdominal pain, in a 9-year-old girl who had a history of aggressive behavior and early anorexia [47]. The patient was diagnosed with coproporphyria – a subtype of porphyria in which psychiatric signs (labile mood and psychosis) have occasionally been described among affected children and young adults [47-51].

### Wilson disease (WD)

#### Key features

WD is an autosomal recessive disorder with a prevalence of 6 per 100 000 of the general population [39]. A mutation in the *ATP7B* gene coding for a key copper transport protein leads to copper accumulation in the liver, brain, kidney and skeletal system, caused by reduced excretion in the bile [52].

In approximately half of patients, computerized tomography reveals characteristic hypodensities in the basal ganglia [53]. Virtually all patients show magnetic resonance imaging (MRI) abnormalities, including T2-weighted hyperintensities in the thalamus, brainstem and lenticular nuclei [54]. Functional imaging generally shows significant hypometabolism in the lenticular nuclei [55].

Classically, symptoms of WD appear between the ages of 6 and 20 years. Approximately one-third of patients initially present with hepatic disease, one-third with neurological symptoms, and one-third with psychiatric symptomatology. Kayser-Fleischer ring is seen in some patients during ophthalmological examinations. Between one- and two-thirds of patients report psychiatric symptoms at initial presentation [56-58]. Psychiatric signs are present in almost 50% of patients at any one time [59], and present before motor signs in 20% of cases; up to half of patients may be seen initially by a psychiatrist [60].

#### Psychiatric signs

Eleven relevant case reports or case series (psychiatric or general including psychiatric patients) were identified, and data from most of these were included in a large review [57]. Four main psychiatric symptom clusters have been identified: mood and affective change; behavior and personality change; psychosis and; cognitive impairment [61]. Personality changes are very common, particularly irritability and aggression [56,62]. Mood disturbance, including both depression and mania, is the most common formal neuropsychiatric illness [63-67]. Psychosis, delusional states and catatonia, while less frequent in WD, can be extremely disabling [56,57,60,61,68]. Schizophrenia-like symptoms were reported to be present in up to 10% of patients [63], but were less prevalent in one case series (2.4%) [69]. Whilst delusions in WD have been reported to be uncommon [60], a number of psychotic presentations meeting criteria for delusional disorder have recently been described [70-73].

Deteriorating academic performance or work function is another key neurological feature of WD. Neurologically symptomatic patients display a range of cognitive difficulties including impairments of executive function, aspects of memory and visuospatial processing [59,74,75]. In contrast, no such deficits are found in neurologically asymptomatic patients [76]. Lesions within the basal ganglia seem to be of central importance in cognitive change due to their interruption of frontal-subcortical circuits [76,77].

After initiating treatment with chelation therapy, the disease often stabilizes or improves, but disease progression during treatment is more likely for neuropsychiatric symptoms than for hepatic symptoms [78]. Resolution of neuropsychiatric illness following chelation has been reported [67,73,79,80].

The use of neuroleptic medication may be problematic due to the increased risk of movement disorder side effects in the setting of degenerative basal ganglia disease [81-84]. However, some reports suggest the relatively safe use of atypical medications such as olanzapine, risperidone, quetiapine and clozapine, which each have a lower propensity to cause movement disorders [72,83,85,86]. Nevertheless, these agents should be used with caution because of the increased risk of agranulocytosis in the presence of hypersplenism or penicillamine treatment.

Treatment of mania with mood stabilizers can be difficult because valproate or carbamazepine may be contraindicated in the presence of significant hepatic impairment [84]. Lithium may also be contraindicated in the presence of renal tubular acidosis [84], although successful lithium treatment without metabolic compromise has been reported [86,87].

Electroconvulsive therapy (ECT) has been successfully used in cases of catatonia [88], psychosis [89] and depression [90-92].

Depression has been reported as responding to both tricyclic antidepressants and selective serotonin reuptake inhibitors [91-93], although treatment-resistance to traditional antidepressants has also been described [91]. A manic switch in response to antidepressant therapy has also been described in one patient [93].

### Cerebrotendinous xanthomatosis (CTX)

#### Key features

CTX is an autosomal recessive disease of bile acid synthesis. It is caused by mutations in the *CYP27A1* gene, which is localized on the long arm of chromosome 2 and codes for the mitochondrial enzyme, sterol-27-hydroxylase. This enzyme is involved in the synthesis of chenodeoxycholic and cholic acids from cholesterol. The metabolic block resulting from the mutant gene causes a progressive storage of cholesterol and its poorly soluble by-product, cholestanol, which is deposited in many tissues including the brain and tendons [94]. A recent review found more than 300 patients with CTX reported worldwide, and identified 50 different mutations in the *CYP27A1* gene associated with this disease [95]. Prevalence is estimated around 2:100 000 [96].

Clinical presentations of CTX are quite variable. The initial symptoms typically begin in childhood with non-specific mild mental retardation, juvenile cataract, chronic diarrhea or epilepsy. Progressive neurological deterioration follows in adolescence or adulthood with acute psychiatric signs [10,97], progressive spastic paraparesis, cerebellar ataxia, polyneuropathy, epilepsy and cognitive deficits leading to severe handicap or death. These neurological signs can be accompanied by the appearance of tendon xanthomata, which are usually visible at the level of the Achilles’ tendons. An MRI of the brain typically shows a specific pattern with high signals in the dentate nuclei of the cerebellum on T2-weighted sequences [98].

Chenodeoxycholic acid is the primary treatment for CTX. This agent blocks the accumulation of cholestanol by replenishing the pool of bile acid in the liver and hepatic circulation, and shuts down the abnormal hepatic bile acid synthesis pathway. Although it is efficient at normalizing circulating levels of cholestanol, and clearly stabilizes disease progression, it does not improve already existing neurological signs. In addition, xanthomata do not decrease in size.

#### Psychiatric signs

Fourteen articles were identified in the systematic literature review [10,97]. Psychiatric manifestations in CTX have only been described in sporadic reports and two patient series [97,99-111]. Unfortunately, many of these cases are poorly documented and do not contain a systematic psychiatric evaluation.

Acute psychotic episodes have been described, but most psychiatric symptoms are non-specific and occur during childhood and/or adolescence [10,97]. Hyperactivity is the most common syndrome seen during youth, and is associated with cognitive impairments in speech and comprehension [112].

The Dotti et al. series described 11/13 patients (85%) with psychiatric symptoms [Note: please confirm that 11 patients out of the 13 studied had psychiatric symptoms, as queried]: five with behavioural changes, four with psychosis and two with depression [107], suggesting an over-representation of psychiatric disorders in this population. This contrasts with the documented rarity of psychiatric signs in CTX (around 10%). In the only small series specifically focusing on the psychiatric spectrum of CTX, Berginer et al. reported four patients with disparate psychiatric syndromes, including irritability and personality changes with hypersexuality, atypical psychosis and paranoid delusions, and severe catatonia [97]. Diagnoses of CTX were made on the basis of pes cavus and Achilles xanthomata in all patients, and caratacts and cognitive impairment in two cases.

Two siblings were recently described with an early psychiatric presentation comprising attention deficit hyperactivity disorder (ADHD) and oppositional defiant disorder (ODD) associated with mild intellectual disability [112]. In both patients, treatment with chenodeoxycholic acid improved externalizing symptoms, and a partial recovery of cognitive impairment was observed.

### Niemann Pick disease type C (NP-C)

#### Key features

NP-C is a pan-ethnic, autosomal recessive neurodegenerative disease with an incidence estimated between 1 case per 150 000 and 1 case per 120 000 live births [113,114]. The disease is characterized by a variety of progressive, disabling neurological symptoms including clumsiness, limb and gait ataxia, dysarthria, dysphagia and cognitive deterioration [113,115].

NP-C is associated with mutations of the *NPC1* and *NPC2* genes, with no primary defect in catabolic enzymes. *NPC1* gene mutations are present in 95% of cases and *NPC2* mutations are present in approximately 4%. At the cellular level, these mutations give rise to characteristic abnormalities in the intracellular transport of cholesterol, glycosphingolipids and sphingosine. Impaired function of the *NPC1* and *NPC2* gene products, which normally function cooperatively in intracellular lipid transport, leads to the accumulation of these lipids in the late-endosomal/lysosomal intracellular compartment, and excess build up in various tissues. Unesterified cholesterol, sphingomyelin, bis(monoacylglycero)-phosphate, glycosphingolipids and sphingosine are stored in excess in the liver and spleen, while levels of glucosylceramide, lactosylceramide and, above all, GM2 and GM3 gangliosides are markedly increased in the brain [116].

NP-C has an extremely heterogeneous clinical presentation characterized by a wide range of symptoms that are not specific to the disease, and which arise and progress over varied periods of time [113,117]. This complicates diagnosis, and is likely an important factor in the under-detection of the NP-C and, in some cases, its misdiagnosis. In the first decade of life, the most common presentations are neurological, although early-onset patients are often diagnosed based on isolated systemic manifestations (e.g. neonatal jaundice, splenomegaly). Many cases are also diagnosed in adulthood, sometimes even up to the seventh decade of life [118].

The age at onset of neurological symptoms has a major influence on disease progression; [119] if neurological symptoms arise early in life the rate of deterioration is generally faster and premature death occurs sooner. Patients with the perinatal-onset form present during the first 3 months of life with an enlarged liver and spleen, prolonged cholestasis, hydrops fetalis and/or respiratory failure [113,114], usually without presenting neurological signs. Infantile, juvenile and adolescent/adult forms usually present with neurological signs including progressive ataxia, dystonia, dysarthria, dysphagia, deafness, cataplexy or, more rarely, epilepsy. Most notably, vertical supranuclear gaze palsy (VSGP) – particularly paresis of downgaze – is a highly specific and highly prevalent sign that may be present at an early stage of the disease [113,115]. VSGP or discrete slowing of saccades is present in almost all cases at some point during the disease course.

Diagnosis of NP-C requires a skin biopsy and a fibroblast culture in a specialized center, with the resulting cultured cells stained with filipin (which binds excess cholesterol) and tested for cholesterol esterification. However, data suggest that plasma oxysterol measurements may represent a simpler screening and/or diagnostic method in the coming years [120].

Therapy for NP-C has, until recently, been limited to supportive measures, including pharmacotherapy to alleviate neurological and psychiatric symptoms [113,115,121]. Miglustat, an iminosugar compound that reversibly inhibits glucosyl ceramide synthetase and thus inhibits the formation of excess gangliosides, is a substrate-reduction therapy that has been shown to stabilize neurological manifestations in children and adults [122-124].

#### Psychiatric signs

Numerous cases of NP-C presenting with schizophrenia-like symptoms have been reported in adolescent and adult patients, and nine case series and reports were identified and included in this literature analysis [125-133]. Definitive diagnoses are commonly delayed in patients with adult psychiatric presentations of NP-C, sometimes by up to 10 years [134].

Psychotic presentations among children and adolescents with NP-C have been reported, and may be comorbid with a pervasive developmental disorder (PDD). Sandu et al. reported a case of an 8-year-old with PDD who presented with auditory hallucinations and 7 years later developed a typical paranoid schizophrenic illness that was partially responsive to risperidone [132]. One notable report described two siblings with psychosis [131]. The male sibling presented at 16 years old with visual and auditory hallucinations, and later developed dysarthria and ataxia leading to a definitive diagnosis at the age of 24 years when vertical supranuclear ophthalmoplegia was discovered. His sister developed schizophrenia-like symptoms a decade later, but diagnosis was made rapidly when she was examined for vertical supranuclear ophthalmoplegia based on the family history. Notably, her later onset and lower antipsychotic dosage required to effect symptom resolution mirrored the gender dimorphism seen in typical schizophrenia, which raises the possibility of a gender effect in presentation and progression of NP-C [131].

The onset of developmental delay is commonly seen between 6 and 15 years of age in NP-C, and may result in a learning disorder and/or impaired school performance [117,135,136]. Patients commonly display cognitive impairments involving logical thinking and abstraction, impaired attentional processes, poor working memory, word retrieval difficulties, and a lack of interpersonal ‘distance’ [121,136]. The typical cognitive profile in adult patients is one of significant executive dysfunction and impaired working memory [115,136].

## Discussion

This article is the first systematic review in this field. Widely unknown and neglected by psychiatrists, IEMs represent a growing field in research that interfaces with clinical psychiatry due to the fact that a number of disorders may initially present to psychiatrists. New treatments are available for a number of these diseases [137].

One key finding of this literature review is that the clinical signs of IEMs are poorly documented in terms of both quantity (only 63 articles with original data for seven different disorders) and quality (non-systematic clinical evaluations, lack of standardized clinical scales, sparse clinical description). A second clear finding is that many cases of WD, MTHFR-D and NP-C are strongly associated with psychotic illness.

It is not realistic, and probably unnecessary due to the rarity of the association, to consider IEMs in all psychiatric patients. It is also not feasible to train psychiatrists to become metabolic specialists. However, it is crucial for all professionals working in psychiatry to be aware of the large variety of organic disorders that may be associated with psychiatric diseases, particularly treatable IEMs such as those addressed in this review, and to be aware of clinical features that may herald an underpinning organic disorder for the patient’s psychiatric presentation.

**One major difficulty** in considering the association of IEMs with psychosis is in ascribing causality. If prevalence rates across a number of populations are higher than 0.8–1.0% (the general population prevalence for schizophrenia) [138,139] it may be difficult to consider an association with IEMs as significant as their prevalence is more likely around 1 in 10 000. In addition, when treatment of an organic disorder leads to an improvement in psychotic symptoms, this association is strengthened, and the Bradford-Hill criteria of strength, consistency and temporality are met [140]. These associations may also aid in shedding light on the potential neurobiological origins of schizophrenia. There is a wide consensus regarding the neurodevelopmental hypothesis of schizophrenia [141,142], as well as the role of complex genetic determinism and gene–environment interactions [143]. The historical association of a range of organic disorders with schizophrenia-like psychosis has shed light on the role that the medial temporal lobe and diencephalon play in the origin of psychotic symptoms [2], and similarly the recent advancement in our understanding of the neurobiology of various IEMs has shed light on the role of anatomical disconnection and disruption to a range of neurotransmitter systems in the genesis of psychotic illness [130].

**Figure 2:**
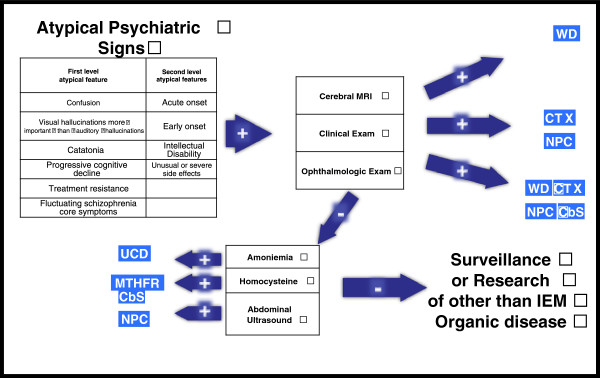
**Diagnostic algorithm for diagnosing inborn errors of metabolism in patients with schizophrenia-like symptoms.** Negative: If exams are negative and suspicion is high. Positive: Could lead to diagnoses or high suspicion of specific disease. MRI = magnetic resonance imaging; MTHFR-CbS = methylenetetrahydrofolate reductase-cystathionine beta-synthase; NP-C = Niemann-Pick disease type C; UCDs = urea cycle disorders, WD = Wilson disease.

**The second major difficulty** is to recognize IEMs and to think about an organic etiology in clinical practice. In order to help psychiatrists, it could be clinically useful to identify psychiatric features that may trigger for the search of organic disorder in patients with schizophrenia. Unfortunately, data regarding organic psychosis and its specific associated symptoms are scarce. One study, which was not specific for IEMs, analyzed the phenomenology of 74 patients with ‘organic schizophrenia’ compared with ‘non-organic schizophrenia’ [144]. Visual hallucinations and confusion were seen more often among patients with organic schizophrenia, and comparable features have been observed in elderly schizophrenia patients [145,146]. A handful of inborn errors of metabolism may cause elementary hallucination and visual hallucinations which are associated with various organic and psychiatric conditions [147]. Hallucinations are a core symptom of schizophrenia and are more often auditory or at least, auditory hallucinations are more important than visual hallucinations. We suggest therefore that predominant visual hallucinations are highly suggestive of organic disorders such as IEM. An acute onset of psychiatric symptoms may also raise suspicion of IEMs (e.g. UCDs, porphyria or homocysteinemia with CbS-D). It is also notable that data indicate a high degree of association of catatonia with organic disorders, especially if it occurs during childhood or adolescence [148]. An unusually high proportion of patients with organic disorders has been reported in large series of patients with early-onset schizophrenia, which suggests that an early-onset of schizophrenia-like symptoms is another indicator for possible organic origin of disease, especially if associated with progressive cognitive decline, which is a common feature in IEMs [149]. Finally, treatment resistance is frequently associated with IEMs [150], again suggesting its possible use as an indicator for possible organic disease. In summary, we may suggest six readily recognizable features that should trigger the suspicion of organicity associated with schizophrenia-like symptoms: 1) acute confusion; 2) visual hallucinations more important than auditory hallucinations; 3) catatonia; 4) progressive cognitive decline; 5) early or acute onset and; 6) treatment resistance (see Table 1). As the validity and specificity of these atypical psychiatric signs have not yet been evaluated, they are presented to raise awareness and suggest clinical and neurological exams prior to further progressive screening. Both atypical psychiatric signs and main clinical/biomarker features of IEM lead us to propose an algorithm (see Figure 2). This algorithm is based on clinical practice of OB, HK and MW and DC. We plan to study his validity and reliance in further study in population of patients with psychiatric signs and IEM. Further research is needed to develop a real suspicion index from our group of atypical psychiatric signs associated with this algorithm.

**Table 1:** Atypical psychiatric features which should trigger a search for inborn error of metabolism in patients with schizophrenia

**First level atypical feature (atypical on their own)**	**Second level atypical features (atypical when associated with first level)**
Confusion	Acute onset
Visual hallucinations more important than auditory hallucinations	Early onset
Catatonia	Intellectual Disability
Progressive cognitive decline	Unusual or severe side effects
Treatment resistance	
Fluctuating schizophrenia core symptoms	

As the validity and specificity of these atypical psychiatric signs have not yet been validated, they are presented to rise suspicion and suggest clinical and neurological exam prior to further progressive screening.

Biological screening is not recommended for all patients but only in case of high suspicion. It is also important to know that the cost of screening for most of these disorders in standard metabolic reference laboratories is usually modest, generally consisting of serum or urine tests for various metabolites, comparable or less than the cost of basic neuroimaging. The costs of treating these IEMs varies widely; from B-vitamin replacement and/or dietary modification in a number of disorders, to more expensive long-term treatments in disorders such as NPC, and potential high-cost hospitalizations during metabolic crises in UCDs and porphyria. This should however be referenced against the cost of treating true schizophrenia, at a cost of at least $USD 50000 per year [151], with at least one third of this borne in direct treatment.

Toward atypical psychiatric signs, IEMs presents specificities and Table 2 provides a summary that encompasses the main clinical signs of six treatable IEMs, and highlights the main clinical symptoms, biomarkers and context as well as ophthalmologic signs, which occur in at least four of the treatable IEMs.

**Table 2:** Synthesis of main clinical, contextual, ophthalmologic symptoms associated with 7 treatable IEM associated with schizophrenia-like symptoms

**Disorder**	**Clinical signs**	**Context**	**Eye exam**	**Biomarkers**
**Wilson disease (WD)**	Tremor	–	Kayser-Fleischer ring	Ceruloplasmin
	Dystonia			
	Dysarthria			
**Urea cycle disorders (UCDs)**	Confusion	Protein diet	–	Hyperammoniemia
	Abdominal pain	Post surgery		
	Nausea/vomiting	Drugs*		
**Homocysteinemia (MTHFR)**	Ataxia	–	–	Homocysteinemia
	Mental regression			Methioninemia
**Homocysteinemia (CbS)**	Thromboembolism	Protein diet	Severe myopia	Homocysteinemia
	Scoliosis	Post surgery	Ectopic lens	Methioninemia
	Marfan-like cerebellar signs			
**Niemann-Pick disease type C (NP-C)**	Dystonia + ataxia Dysarthria	Neonatal icterus	Supranuclear vertical	Skin biopsy
	Splenomegaly	Slow progression	gaze palsy	Filipin test
				*NPC1* and *NPC2* gene test
**Cerebrotendinous xanthomatosis (CTX)**	Chronic diarrhea	–	Juvenile cataract	High cholestanol
	Spastic paralysis			
**Porphyria (POR)**	Black or red urine	Periodic	–	Porphobilinogens (URINE)
	Constipation			
	Confusion			
	Abdominal pain			
	Nausea/vomiting			

*Example drugs: valproate/corticoids.

It is important for psychiatrists and other associated professionals to be specifically aware of potential IEMs when patients present with such indicators (organic signs and atypical psychiatric symptoms) of possible organic psychosis.

## Conclusions

Based on published evidence this review highlights the role of a range of IEMs as possible underlying organic causes of schizophrenia or schizophrenia-like syndromes. It is important to identify such cases as some IEMs are treatable (sometimes simply with vitamin replacement or supplementation) and new treatments continue to appear. Clinical studies suggest that some IEM-specific treatments may be most effective during the early stages of disease when psychiatric symptoms may be evident. Efficient recognition and identification of the underlying organic disease could therefore allow earlier initiation of specific therapy and, possibly, improve outcomes.Although the literature base from which to draw conclusions is limited, clinicians managing patients presenting with new-onset psychosis should pay particular attention to IEMs as a possible underlying cause in patients with atypical symptoms, and in the presence of specific clinical contexts. It is hoped that this review summarizing six easily assessed features that might trigger suspicion of organicity in patients with psychosis will help to detect patients with treatable IEMs as early as possible during their disease course. While not intended to replace specialized psychiatric or neurological examination and measurements, our proposed algorithm (Figure 2) is a pragmatic tool that can be used to reduce the risk of mistaken diagnoses among patients with atypical psychiatric signs and treatable IEMs.

## Competing interest

OB, MW, FS and HK declare past and present honorarium from Actelion Pharma. DC and ST have no competing interest.

## Authors’ contribution

OB write the first draft of the article which was reviewed by all authors. ST, DC, MW and HK help OB for response to reviewers. Regarding specific systematic review work, two groups worked separately in screening abstracts from relevant articles from the literature review (Group 1: MW and HK. Group 2: OB, DC, ST and FS). All authors read and approved the final manuscript.

## References

[B1] Price G Ron MA Schapira AHV Schizophrenia and schizophrenia-like psychosis Neurology and Clinical Neuroscience 2003 1 St Louis: Elsevier-Mosby 223 233

[B2] Davison K Bagley CR Schizophrenia-like psychoses associated with organic cerebral disorders: a review Psychiatr Dev 1983 1 1 1 33 6232606

[B3] Johnstone EC Macmillan JF Crow TJ The occurrence of organic disease of possible or probable aetiological significance in a population of 268 cases of first episode schizophrenia Psychol Med 1987 17 2 371 379 3602229 10.1017/s0033291700024922

[B4] Lauterbach MD Stanislawski-Zygaj AL Benjamin S The differential diagnosis of childhood and young adult onset disorders that include psychosis J Neuropsychiatry Clin Neurosci 2008 20 4 409 418 19196925 10.1176/jnp.2008.20.4.409

[B5] Saudubray JM Neurometabolic disorders J Inherit Metab Dis 2009 32 5 595 596 19757144 10.1007/s10545-009-9958-9

[B6] Applegarth DA Toone JR Lowry RB Incidence of inborn errors of metabolism in British Columbia, 1969–1996 Pediatrics 2000 105 1 e10 10617747 10.1542/peds.105.1.e10

[B7] Sedel F Baumann N Turpin JC Lyon-Caen O Saudubray JM Cohen D Psychiatric manifestations revealing inborn errors of metabolism in adolescents and adults J Inherit Metab Dis 2007 30 5 631 641 17694356 10.1007/s10545-007-0661-4

[B8] Wilson WP Nashold BS Jr Psychiatric considerations of certain neurological diseases treated neurosurgically Int Psychiatry Clin 1967 4 2 189 204 4230190

[B9] Bonnot O Cohen D [Psychiatric and cognitive signs associated with inborn errors of metabolism] Rev Neurol 2011 167 12 881 885 22041824 10.1016/j.neurol.2011.03.014

[B10] Estrov Y Scaglia F Bodamer OA Psychiatric symptoms of inherited metabolic disease J Inherit Metab Dis 2000 23 1 2 6 10682302 10.1023/a:1005685010766

[B11] Martins AM Inborn errors of metabolism: a clinical overview. Sao Paulo medical journal = Rev Paul Med 1999 117 6 251 265 10.1590/s1516-31801999000600006 10625889

[B12] Trifiletti RR Packard AM Metabolic disorders presenting with behavioral symptoms in the school-aged child Child Adolesc Psychiatr Clin N Am 1999 8 4 791 806 10553204

[B13] KEGG Reference Metabolic Pathways 2013 [cited 2014 January]; Available from: http://www.genome.jp/kegg/pathway.html

[B14] Yap S 2007 [cited January, 2014]; Available from: http://www.orpha.net/consor/cgi-bin/OC_Exp.php?Lng=FR&Expert=394

[B15] Abbott MH Folstein SE Abbey H Pyeritz RE Psychiatric manifestations of homocystinuria due to cystathionine beta-synthase deficiency: prevalence, natural history, and relationship to neurologic impairment and vitamin B6-responsiveness Am J Med Genet 1987 26 4 959 969 3591841 10.1002/ajmg.1320260427

[B16] Li SC Stewart PM Homocystinuria and psychiatric disorder: a case report Pathology 1999 31 3 221 224 10503267 10.1080/003130299105025

[B17] Baric I Inherited disorders in the conversion of methionine to homocysteine J Inherit Metab Dis 2009 32 4 459 471 19585268 10.1007/s10545-009-1146-4

[B18] Mattson MP Shea TB Folate and homocysteine metabolism in neural plasticity and neurodegenerative disorders Trends Neurosci 2003 26 3 137 146 12591216 10.1016/S0166-2236(03)00032-8

[B19] Roze E Gervais D Demeret S Ogier de Baulny H Zittoun J Benoist JF Said G Pierrot-Deseilligny C Bolgert F Neuropsychiatric disturbances in presumed late-onset cobalamin C disease Arch Neurol 2003 60 10 1457 1462 14568819 10.1001/archneur.60.10.1457

[B20] Gilbody S Lewis S Lightfoot T Methylenetetrahydrofolate reductase (MTHFR) genetic polymorphisms and psychiatric disorders: a HuGE review Am J Epidemiol 2007 165 1 1 13 17074966 10.1093/aje/kwj347

[B21] Ellingrod VL Miller DD Taylor SF Moline J Holman T Kerr J Metabolic syndrome and insulin resistance in schizophrenia patients receiving antipsychotics genotyped for the methylenetetrahydrofolate reductase (MTHFR) 677C/T and 1298A/C variants Schizophr Res 2008 98 1–3 47 54 17976958 10.1016/j.schres.2007.09.030 PMC2271139

[B22] Frankenburg FR The role of one-carbon metabolism in schizophrenia and depression Harv Rev Psychiatry 2007 15 4 146 160 17687709 10.1080/10673220701551136

[B23] Friso S Choi SW Gene-nutrient interactions in one-carbon metabolism Curr Drug Metab 2005 6 1 37 46 15720206 10.2174/1389200052997339

[B24] Sharma RP Schizophrenia, epigenetics and ligand-activated nuclear receptors: a framework for chromatin therapeutics Schizophr Res 2005 72 2–3 79 90 15560954 10.1016/j.schres.2004.03.001

[B25] Regland B Schizophrenia and single-carbon metabolism Prog Neuro-Psychopharmacol Biol Psychiatry 2005 29 7 1124 1132 10.1016/j.pnpbp.2005.06.023 16095790

[B26] O'Donnell C Stephens T The significance of homocysteine levels in schizophrenia Am J Psychiatry 2005 162 7 1387 1388 author reply 1388–1389 15994731 10.1176/appi.ajp.162.7.1387-a

[B27] Muntjewerff JW Kahn RS Blom HJ den Heijer M Homocysteine, methylenetetrahydrofolate reductase and risk of schizophrenia: a meta-analysis Mol Psychiatry 2006 11 2 143 149 16172608 10.1038/sj.mp.4001746

[B28] Haberle J Boddaert N Burlina A Chakrapani A Dixon M Huemer M Karall D Martinelli D Crespo PS Santer R Servais A Valayannopoulos V Lindner M Rubio V Dionisi-Vici C Suggested guidelines for the diagnosis and management of urea cycle disorders Orphanet J Rare Dis 2012 7 32 22642880 10.1186/1750-1172-7-32 PMC3488504

[B29] Singh RH Nutritional management of patients with urea cycle disorders J Inherit Metab Dis 2007 30 6 880 887 18034368 10.1007/s10545-007-0718-4

[B30] Smith W Kishnani PS Lee B Singh RH Rhead WJ Sniderman King L Smith M Summar M Urea cycle disorders: clinical presentation outside the newborn period Crit Care Clin 2005 21 4 Suppl S9 17 16227115 10.1016/j.ccc.2005.05.007

[B31] Arn PH Hauser ER Thomas GH Herman G Hess D Brusilow SW Hyperammonemia in women with a mutation at the ornithine carbamoyltransferase locus. A cause of postpartum coma N Engl J Med 1990 322 23 1652 1655 2342525 10.1056/NEJM199006073222307

[B32] Enns GM O'Brien WE Kobayashi K Shinzawa H Pellegrino JE Postpartum “psychosis” in mild argininosuccinate synthetase deficiency Obstet Gynecol 2005 105 5 Pt 2 1244 1246 15863597 10.1097/01.AOG.0000157769.90230.24

[B33] Bachmann C Outcome and survival of 88 patients with urea cycle disorders: a retrospective evaluation Eur J Pediatr 2003 162 6 410 416 12684900 10.1007/s00431-003-1188-9

[B34] Krivitzky L Babikian T Lee HS Thomas NH Burk-Paull KL Batshaw ML Intellectual, adaptive, and behavioral functioning in children with urea cycle disorders Pediatr Res 2009 66 1 96 101 19287347 10.1203/PDR.0b013e3181a27a16 PMC2746951

[B35] Legras A Labarthe F Maillot F Garrigue MA Kouatchet A Ogier de Baulny H Late diagnosis of ornithine transcarbamylase defect in three related female patients: polymorphic presentations Crit Care Med 2002 30 1 241 244 11902270 10.1097/00003246-200201000-00035

[B36] Myers JH Shook JE Vomiting, ataxia, and altered mental status in an adolescent: late-onset ornithine transcarbamylase deficiency Am J Emerg Med 1996 14 6 553 557 8857803 10.1016/S0735-6757(96)90097-2

[B37] Panlaqui OM Tran K Johns A McGill J White H Acute hyperammonemic encephalopathy in adult onset ornithine transcarbamylase deficiency Intensive Care Med 2008 34 10 1922 1924 18651132 10.1007/s00134-008-1217-2

[B38] Thurlow VR Asafu-Adjaye M Agalou S Rahman Y Fatal ammonia toxicity in an adult due to an undiagnosed urea cycle defect: under-recognition of ornithine transcarbamylase deficiency Ann Clin Biochem 2010 47 Pt 3 279 281 20406775 10.1258/acb.2010.009250

[B39] Orphanet Prevalence of rare diseases by alphabetical list 2013 [cited January, 2014]; Available from: http://www.orpha.net/orphacom/cahiers/docs/GB/Prevalence_of_rare_diseases_by_alphabetical_list.pdf

[B40] Cashman MD Psychiatric aspects of acute porphyria Lancet 1961 1 7168 115 116 13691283 10.1016/s0140-6736(61)92169-9

[B41] Tishler PV Woodward B O'Connor J Holbrook DA Seidman LJ Hallett M Knighton DJ High prevalence of intermittent acute porphyria in a psychiatric patient population Am J Psychiatry 1985 142 12 1430 1436 4073306 10.1176/ajp.142.12.1430

[B42] Boon FF Ellis C Acute intermittent porphyria in a children’s psychiatric hospital J Am Acad Child Adolesc Psychiatry 1989 28 4 606 609 2768156 10.1097/00004583-198907000-00022

[B43] Santosh PJ Malhotra S Varied psychiatric manifestations of acute intermittent porphyria Biol Psychiatry 1994 36 11 744 747 7858070 10.1016/0006-3223(94)90085-x

[B44] Goldberg A Acute intermittent porphyria: a study of 50 cases Q J Med 1959 28 110 183 209 13658350

[B45] Stein JA Tschudy DP Acute intermittent porphyria. A clinical and biochemical study of 46 patients Medicine 1970 49 1 1 16 4907358

[B46] Bonkowsky HL Schady W Neurologic manifestations of acute porphyria Semin Liver Dis 1982 2 2 108 124 6753161 10.1055/s-2008-1040701

[B47] Mandoki MW Sumner GS Psychiatric manifestations of hereditary coproporphyria in a child J Nerv Ment Dis 1994 182 2 117 118 8308532 10.1097/00005053-199402000-00012

[B48] Brodie MJ Thompson GG Moore MR Beattie AD Goldberg A Hereditary coproporphyria. Demonstration of the abnormalities in haem biosynthesis in peripheral blood Q J Med 1977 46 182 229 241 866576

[B49] Crimlisk HL The little imitator–porphyria: a neuropsychiatric disorder J Neurol Neurosurg Psychiatry 1997 62 4 319 328 9120442 10.1136/jnnp.62.4.319 PMC1074085

[B50] Kuhnel A Gross U Doss MO Hereditary coproporphyria in Germany: clinical-biochemical studies in 53 patients Clin Biochem 2000 33 6 465 473 11074238 10.1016/s0009-9120(00)00159-4

[B51] Gross U Puy H Meissauer U Lamoril J Deybach JC Doss M Nordmann Y Doss MO A molecular, enzymatic and clinical study in a family with hereditary coproporphyria J Inherit Metab Dis 2002 25 4 279 286 12227458 10.1023/a:1016598207397

[B52] Pfeffer RF Wilson’s disease Semin Neurol 2007 27 2 123 132 17390257 10.1055/s-2007-971173

[B53] Williams J Walshe J Wilson’s disease: an analysis of the cranial computerized tomographic appearances found in 60 patients and the changes in response to treatment with chelating agents Brain 1981 104 735 752 7326565 10.1093/brain/104.4.735

[B54] Roh JK Lee TG Wie BA Lee SB Park SH Chang KH Initial and follow-up brain MRI findings and correlation with the clinical course in Wilson’s disease Neurology 1994 44 6 1064 1068 8208401 10.1212/wnl.44.6.1064

[B55] Hawkins RA Mazziotta JC Phelps ME Wilson’s disease studied with FDG and positron emission tomography Neurology 1987 37 11 1707 1711 3499583 10.1212/wnl.37.11.1707

[B56] Barthel W Markwardt F Aggregation of blood platelets by adrenaline and its uptake Biochem Pharmacol 1975 24 20 1903 1904 20 10.1016/0006-2952(75)90415-3

[B57] Dening TR The neuropsychiatry of Wilson’s disease: a review Int J Psychiatry Med 1991 21 2 135 148 1894453 10.2190/BAFK-D0A7-Q1C4-V667

[B58] Schwartz M Fuchs S Polak H Sharf B [Psychiatric manifestations in Wilson’s disease] Harefuah 1993 124 2 75 77 120 8436325

[B59] Rathbun JK Neuropsychological aspects of Wilson’s disease Int J Neurosci 1996 85 3–4 221 229 8734560 10.3109/00207459608986684

[B60] Dening TR Berrios GE Wilson’s disease. Psychiatric symptoms in 195 cases Arch Gen Psychiatry 1989 46 12 1126 1134 2589927 10.1001/archpsyc.1989.01810120068011

[B61] Dening TR Psychiatric aspects of Wilson’s disease Br J Psychiatry 1985 147 677 682 3830328 10.1192/bjp.147.6.677

[B62] Coscia L Causa P Giuliani E Nunziata A Pharmacological properties of new neuroleptic compounds Arzneimittelforschung 1975 25 9 1436 1442 25

[B63] Akil M Brewer GJ Psychiatric and behavioral abnormalities in Wilson’s disease Adv Neurol 1995 65 171 178 7872138

[B64] Akil M Schwartz JA Dutchak D Yuzbasiyan-Gurkan V Brewer GJ The psychiatric presentations of Wilson’s disease J Neuropsychiatry Clin Neurosci 1991 3 4 377 382 1821256 10.1176/jnp.3.4.377

[B65] Dening TR Berrios GE Wilson’s disease: a longitudinal study of psychiatric symptoms Biol Psychiatry 1990 28 3 255 265 2378928 10.1016/0006-3223(90)90581-l

[B66] Medalia A Scheinberg IH Psychopathology in patients with Wilson’s disease Am J Psychiatry 1989 146 5 662 664 2712173 10.1176/ajp.146.5.662

[B67] Srinivas K Sinha S Taly AB Prashanth LK Arunodaya GR Janardhana Reddy YC Khanna S Dominant psychiatric manifestations in Wilson’s disease: a diagnostic and therapeutic challenge! J Neurol Sci 2008 266 1–2 104 108 17904160 10.1016/j.jns.2007.09.009

[B68] Renaud B Buda M Lewis BD Pujol JF Effects of 5,6-dihydroxytryptamine on tyrosine-hydroxylase activity in central catecholaminergic neurons of the rat Biochem Pharmacol 1975 24 18 1739 1742 17 10.1016/0006-2952(75)90018-0

[B69] Taly AB Meenakshi-Sundaram S Sinha S Swamy HS Arunodaya GR Wilson disease: description of 282 patients evaluated over 3 decades Medicine 2007 86 2 112 121 17435591 10.1097/MD.0b013e318045a00e

[B70] Sagawa M Takao M Nogawa S Mizuno M Murata M Amano T Koto A [Wilson’s disease associated with olfactory paranoid syndrome and idiopathic thrombocytopenic purpura] No to shinkei = Brain and nerve 2003 55 10 899 902 14635519

[B71] Wichowicz H Cubala W Slawek J Wilson’s disease asociated with delusional disorder Psychiatry Clin Neurosci 2006 60 758 760 17109711 10.1111/j.1440-1819.2006.01592.x

[B72] Spyridi S Diakogiannis I Michaelides M Sokolaki S Iacovides A Kaprinis G Delusional disorder and alcohol abuse in a patient with Wilson’s disease Gen Hosp Psychiatry 2008 30 6 585 586 19061688 10.1016/j.genhosppsych.2008.05.005

[B73] Stiller P Kassubek J Schonfeldt-Leucona C Connemann B Wilson’s disease in psychiatric patients Psychiatry Clin Neurosci 2008 56 649 12485310 10.1046/j.1440-1819.2002.01071.x

[B74] Medalia A Isaacs-Glaberman K Scheinberg IH Neuropsychological impairment in Wilson’s disease Arch Neurol 1988 45 5 502 504 3358700 10.1001/archneur.1988.00520290030009

[B75] Isaacs-Glaberman K Medalia A Scheinberg IH Verbal recall and recognition abilities in patients with Wilson’s disease Cortex 1989 25 3 353 361 2805722 10.1016/s0010-9452(89)80050-4

[B76] Ardenne M Reitnauer PG Demonstration of tumor inhibiting properties of a strongly immunostimulating low-molecular weight substance. Comparative studies with ifosfamide on the immuno-labile DS carcinosarcoma. Stimulation of the autoimmune activity for approx. 20 days by BA 1, a N-(2-cyanoethylene)-urea. Novel prophylactic possibilities Arzneimittelforschung 1975 25 9 1369 1379 22

[B77] Kroger H Donner I Skiello G Influence of a new virostatic compound on the induction of enzymes in rat liver Arzneimittelforschung 1975 25 9 1426 1429 24

[B78] Smith RJ Bryant RG Metal substitutions incarbonic anhydrase: a halide ion probe study Biochem Biophys Res Commun 1975 66 4 1281 1286 3 10.2196/48138 PMC10704303

[B79] Walter G Lyndon B Depression in hepatolenticular degeneration (Wilson’s disease) Aust N Z J Psychiatry 1997 31 6 880 882 9483264 10.3109/00048679709065517

[B80] Machado A Deguti M Caixeta L Spitz M Lucato L Barbosa E Mania as the first manifestation of Wilson’s disease Bipolar Disord 2008 10 447 450 18402634 10.1111/j.1399-5618.2007.00531.x

[B81] Tu J The inadvisability of neuroleptic medication in Wilson’s disease Biol Psychiatry 1981 16 10 963 968 6118185

[B82] Hoogenraad T Wilson’s Disease 1996 London: Saunders

[B83] Chroni E Lekka NP Tsibri E Economou A Paschalis C Acute, progressive akinetic-rigid syndrome induced by neuroleptics in a case of Wilson’s disease J Neuropsychiatry Clin Neurosci 2001 13 4 531 532 11748326 10.1176/jnp.13.4.531

[B84] Varghese ST Narayanan D Dinesh D Mania in a patient with Wilson’s disease awaiting liver transplant J Neuropsychiatry Clin Neurosci 2008 20 4 501 502 19196945 10.1176/jnp.2008.20.4.501a

[B85] Krim E Barroso B [Psychiatric disorders treated with clozapine in a patient with Wilson’s disease] Presse Med 2001 30 15 738 11360740

[B86] Kulaksizoglu IB Polat A Quetiapine for mania with Wilson’s disease Psychosomatics 2003 44 5 438 439 12954924 10.1176/appi.psy.44.5.438

[B87] Longanathan S Nayak R Sinha S Taly A Math S Varghese M Treating mania in Wilson’s disease with lithium J Neuropsychiatry Clin Neurosci 2008 20 487 489 19196936 10.1176/jnp.2008.20.4.487

[B88] Rodrigues AC Dalgalarrondo P Banzato CE Successful ECT in a patient with a psychiatric presentation of Wilson’s disease J ECT 2004 20 1 55 15088004 10.1097/00124509-200403000-00017

[B89] Shah N Kumar D Wilson’s disease, psychosis, and ECT Convuls Ther 1997 13 4 278 279 9437572

[B90] Negro P Louza Neto M Results of ECT for a case of depression in Wilson disease J Neuropsychiatry Clin Neurosci 1995 7 384 7580202 10.1176/jnp.7.3.384

[B91] Sechi G Antonio Cocco G Errigo A Deiana L Rosati G Agnetti V Stephen Paulus K Mario Pes G Three sisters with very-late-onset major depression and parkinsonism Parkinsonism Relat Disord 2007 13 2 122 125 16737839 10.1016/j.parkreldis.2006.03.009

[B92] Chan K Cheung R Au-Yeung K Mak W Cheng T Ho S Wilson’s disease with depression and parkinsonism J Clin Neurosci 2004 12 303 305 15851088 10.1016/j.jocn.2004.09.005

[B93] Keller R Torta R Lagget M Crasto S Bergamasco B Psychiatric symptoms as late onset of Wilson’s disease: neuroradiological findings, clinical features and treatment Ital J Neurol Sci 1999 20 49 54 10933485 10.1007/s100720050010

[B94] Moghadasian MH Salen G Frohlich JJ Scudamore CH Cerebrotendinous xanthomatosis: a rare disease with diverse manifestations Arch Neurol 2002 59 4 527 529 11939886 10.1001/archneur.59.4.527

[B95] Gallus GN Dotti MT Federico A Clinical and molecular diagnosis of cerebrotendinous xanthomatosis with a review of the mutations in the CYP27A1 gene Neurol Sci 2006 27 2 143 149 16816916 10.1007/s10072-006-0618-7

[B96] Orphanet Prevalence of Rare Diseases 2013 [cited 2014 January]; Available from: http://www.orpha.net/orphacom/cahiers/docs/FR/Prevalence_des_maladies_rares_par_prevalence_decroissante_ou_cas.pdf

[B97] Berginer VM Foster NL Sadowsky M Townsend JA 3rd Siegel GJ Salen G Psychiatric disorders in patients with cerebrotendinous xanthomatosis Am J Psychiatry 1988 145 3 354 357 3344851 10.1176/ajp.145.3.354

[B98] Barkhof F Verrips A Wesseling P van Der Knaap MS van Engelen BG Gabreels FJ Keyser A Wevers RA Valk J Cerebrotendinous xanthomatosis: the spectrum of imaging findings and the correlation with neuropathologic findings Radiology 2000 217 3 869 876 11110956 10.1148/radiology.217.3.r00dc03869

[B99] Philippart M Van Bogaert L Cholestanolosis (cerebrotendinous xanthomatosis). A follow-up study on the original family Arch Neurol 1969 21 6 603 610 5355255 10.1001/archneur.1969.00480180059004

[B100] Shapiro S Depression in a patient with dementia secondary to cerebrotendinous xanthomatosis J Nerv Ment Dis 1983 171 9 568 571 6886686 10.1097/00005053-198309000-00008

[B101] Burnstein M Buckwalter KA Martel W McClatchey KD Quint D Case report 427: Cerebrotendinous xanthomatosis Skelet Radiol 1987 16 4 346 349 10.1007/BF00361481 3616675

[B102] Laurent A Dairou F Luc G Truffert J Lapresle J de Gennes JL Van Bogaert’s cerebrotendinous xanthomatosis. A study of 3 cases Ann Med Interne 1988 139 6 395 402 3066249

[B103] Wevers RA Cruysberg JR Van Heijst AF Janssen-Zijlstra FS Renier WO Van Engelen BG Tolboom JJ Paediatric cerebrotendinous xanthomatosis J Inherit Metab Dis 1992 15 3 374 376 1405473 10.1007/BF02435980

[B104] Soffer D Benharroch D Berginer V The neuropathology of cerebrotendinous xanthomatosis revisited: a case report and review of the literature Acta Neuropathol 1995 90 2 213 220 7484100 10.1007/BF00294324

[B105] Verrips A Steenbergen-Spanjers GC Luyten JA van den Heuvel LP Keyser A Gabreels FJ Wevers RA Two new mutations in the sterol 27-hydroxylase gene in two families lead to cerebrotendinous xanthomatosis Hum Genet 1996 98 6 735 737 8931710 10.1007/s004390050294

[B106] Sperhake JP Matschke J Orth U Gal A Puschel K Sudden death due to cerebrotendinous xanthomatosis confirmed by mutation analysis Int J Legal Med 2000 113 2 110 113 10741487 10.1007/pl00007709

[B107] Dotti MT Rufa A Federico A Cerebrotendinous xanthomatosis: heterogeneity of clinical phenotype with evidence of previously undescribed ophthalmological findings J Inherit Metab Dis 2001 24 7 696 706 11804206 10.1023/a:1012981019336

[B108] Lee Y Lin PY Chiu NM Chang WN Wen JK Cerebrotendinous xanthomatosis with psychiatric disorders: report of three siblings and literature review Chang Gung Med J 2002 25 5 334 340 12141707

[B109] Guyant-Marechal L Verrips A Girard C Wevers RA Zijlstra F Sistermans E Vera P Campion D Hannequin D Unusual cerebrotendinous xanthomatosis with fronto-temporal dementia phenotype Am J Med Genet A 2005 139A 2 114 117 16278884 10.1002/ajmg.a.30797

[B110] Price Evans DA Salah KA Mobrad MA Mitchell WD Olin M Eggertsen G Cerebrotendinous xanthomatosis in a Saudi Arabian family-genotyping and long-term follow-up Saudi Med J 2007 28 7 1113 1118 17603722

[B111] Gonzalez-Cuyar LF Hunter B Harris PL Perry G Smith MA Castellani RJ Cerebrotendinous xanthomatosis: case report with evidence of oxidative stress Redox Rep 2007 12 3 119 124 17623518 10.1179/135100007X200173

[B112] Bonnot O Fraidakis MJ Lucanto R Chauvin D Kelley N Plaza M Dubourg O Lyon-Caen O Sedel F Cohen D Cerebrotendinous xanthomatosis presenting with severe externalized disorder: improvement after one year of treatment with chenodeoxycholic Acid CNS Spectr 2010 15 4 231 236 20414172 10.1017/s1092852900000067

[B113] Wraith JE Baumgartner MR Bembi B Covanis A Levade T Mengel E Pineda M Sedel F Topcu M Vanier MT Widner H Wijburg FA Patterson MC Group N-CGW Recommendations on the diagnosis and management of Niemann-Pick disease type C Mol Genet Metab 2009 98 1–2 152 165 19647672 10.1016/j.ymgme.2009.06.008

[B114] Vanier MT Niemann-Pick disease type C Orphanet J Rare Dis 2010 5 16 20525256 10.1186/1750-1172-5-16 PMC2902432

[B115] Patterson MC Hendriksz CJ Walterfang M Sedel F Vanier MT Wijburg F on behalf of the NPCGWG Recommendations for the diagnosis and management of Niemann-Pick disease type C: An update Mol Genet Metab 2012 106 3 330 344 22572546 10.1016/j.ymgme.2012.03.012

[B116] Vanier MT Lipid changes in Niemann-Pick disease type C brain: personal experience and review of the literature Neurochem Res 1999 24 4 481 489 10227680 10.1023/a:1022575511354

[B117] Sevin M Lesca G Baumann N Millat G Lyon-Caen O Vanier MT Sedel F The adult form of Niemann-Pick disease type C Brain 2007 130 Pt 1 120 133 17003072 10.1093/brain/awl260

[B118] Trendelenburg G Vanier MT Maza S Millat G Bohner G Munz DL Zschenderlein R Niemann-Pick type C disease in a 68-year-old patient J Neurol Neurosurg Psychiatry 2006 77 8 997 998 16844962 10.1136/jnnp.2005.086785 PMC2077625

[B119] Wraith JE Guffon N Rohrbach M Hwu WL Korenke GC Bembi B Luzy C Giorgino R Sedel F Natural history of Niemann-Pick disease type C in a multicentre observational retrospective cohort study Mol Genet Metab 2009 98 3 250 254 19616462 10.1016/j.ymgme.2009.06.009

[B120] Porter FD Scherrer DE Lanier MH Langmade SJ Molugu V Gale SE Olzeski D Sidhu R Dietzen DJ Fu R Wassif CA Yanjanin NM Marso SP House J Vite C Schaffer JE Ory DS Cholesterol oxidation products are sensitive and specific blood-based biomarkers for Niemann-Pick C1 disease Sci Transl Med 2010 2 56 56ra81 10.1126/scitranslmed.3001417 PMC3170139 21048217

[B121] Patterson MC Platt F Therapy of Niemann-Pick disease, type C Biochim Biophys Acta 2004 1685 1–3 77 82 15465428 10.1016/j.bbalip.2004.08.013

[B122] Patterson MC Vecchio D Prady H Abel L Wraith JE Miglustat for treatment of Niemann-Pick C disease: a randomised controlled study Lancet Neurol 2007 6 9 765 772 17689147 10.1016/S1474-4422(07)70194-1

[B123] Wraith JE Vecchio D Jacklin E Abel L Chadha-Boreham H Luzy C Giorgino R Patterson MC Miglustat in adult and juvenile patients with Niemann-Pick disease type C: long-term data from a clinical trial Mol Genet Metab 2010 99 4 351 357 20045366 10.1016/j.ymgme.2009.12.006

[B124] Patterson MC Vecchio D Jacklin E Abel L Chadha-Boreham H Luzy C Giorgino R Wraith JE Long-term miglustat therapy in children with Niemann-Pick disease type C J Child Neurol 2010 25 3 300 305 19822772 10.1177/0883073809344222

[B125] Shulman LM Lang AE Jankovic J David NJ Weiner WJ Case 1, 1995: psychosis, dementia, chorea, ataxia, and supranuclear gaze dysfunction Mov Disord 1995 10 3 257 262 7651440 10.1002/mds.870100304

[B126] Campo JV Stowe R Slomka G Byler D Gracious B Psychosis as a presentation of physical disease in adolescence: a case of Niemann-Pick disease, type C Dev Med Child Neurol 1998 40 2 126 129 9489503 10.1111/j.1469-8749.1998.tb15374.x

[B127] Turpin JC Baumann N [Presenting psychiatric and cognitive disorders in adult neurolipidoses] Rev Neurol 2003 159 6–7 Pt 1 637 647 12910071

[B128] Josephs KA Van Gerpen MW Van Gerpen JA Adult onset Niemann-Pick disease type C presenting with psychosis J Neurol Neurosurg Psychiatry 2003 74 4 528 529 12640083 10.1136/jnnp.74.4.528 PMC1738356

[B129] Tyvaert L Stojkovic T Cuisset JM Vanier MT Turpin JC De Seze J Vermersch P [Presentation of Niemann-Pick type C disease with psychiatric disturbance in an adult] Rev Neurol 2005 161 3 318 322 15800453 10.1016/s0035-3787(05)85038-6

[B130] Walterfang M Fietz M Fahey M Sullivan D Leane P Lubman DI Velakoulis D The neuropsychiatry of Niemann-Pick type C disease in adulthood J Neuropsychiatry Clin Neurosci 2006 18 2 158 170 16720792 10.1176/jnp.2006.18.2.158

[B131] Walterfang M Fietz M Abel L Bowman E Mocellin R Velakoulis D Gender dimorphism in siblings with schizophrenia-like psychosis due to Niemann-Pick disease type C J Inherit Metab Dis 2009 Sup1 221 226 10.1007/s10545-009-1173-1 19609713

[B132] Sandu S Jackowski-Dohrmann S Ladner A Haberhausen M Bachmann C Niemann-Pick disease type C1 presenting with psychosis in an adolescent male Eur Child Adolesc Psychiatry 2009 18 9 583 585 19267177 10.1007/s00787-009-0010-2

[B133] Walterfang M Kornberg A Adams S Fietz M Velakoulis D Post-ictal psychosis in adolescent Niemann-Pick disease type C J Inherit Metab Dis 2010 13 Sup3 63 65 10.1007/s10545-009-9021-x 20069374

[B134] Klunemann HH Santosh PJ Sedel F Treatable metabolic psychosis that go undetecd: What Niemann Pick type C can teach us Int J Psychiatry Clin Pract 2012 16 3 1 8 22746831 10.3109/13651501.2012.687451

[B135] van de Vlasakker CJ Gabreels FJ Wijburg HC Wevers RA Clinical features of Niemann-Pick disease type C. An example of the delayed onset, slowly progressive phenotype and an overview of recent literature Clin Neurol Neurosurg 1994 96 2 119 123 7924073 10.1016/0303-8467(94)90044-2

[B136] Klarner B Klunemann HH Lurding R Aslanidis C Rupprecht R Neuropsychological profile of adult patients with Niemann-Pick C1 (NPC1) mutations J Inherit Metab Dis 2007 30 1 60 67 17160616 10.1007/s10545-006-0417-6

[B137] Talele SS Xu K Pariser AR Braun MM Farag-El-Massah S Phillips MI Thompson BH Cote TR Therapies for inborn errors of metabolism: what has the orphan drug act delivered? Pediatrics 2010 126 1 101 106 20566615 10.1542/peds.2009-3246

[B138] Andreasen NC The evolving concept of schizophrenia: from Kraepelin to the present and future Schiz Res 1997 28 2–3 105 109 10.1016/s0920-9964(97)00112-6 9468346

[B139] van Os J Driessen G Gunther N Delespaul P Neighbourhood variation in incidence of schizophrenia. Evidence for person-environment interaction Br J Psychiatry 2000 176 243 258 10755071 10.1192/bjp.176.3.243

[B140] Hill AB The Environment and Disease: Association or Causation? Proc R Soc Med 1965 58 295 300 14283879 10.1177/003591576505800503 PMC1898525

[B141] Weinberger DR From neuropathology to neurodevelopment Lancet 1995 346 8974 552 557 7544856 10.1016/s0140-6736(95)91386-6

[B142] Van Gorkom HJ Pulles MP Wessels JS Light-induced changes of absorbance and electron spin resonance in small photosystem II particles Biochim Biophys Acta 1975 408 3 331 339 62 10.1016/0005-2728(75)90134-6

[B143] van Os J Kapur S Schizophrenia Lancet 2009 374 9690 635 645 19700006 10.1016/S0140-6736(09)60995-8

[B144] Cutting J The phenomenology of acute organic psychosis. Comparison with acute schizophrenia Br J Psychiatry 1987 151 324 332 3427288 10.1192/bjp.151.3.324

[B145] Horiguchi J Miyaoka T Shinno H Pathogenesis and symptomatology of hallucinations (delusions) of organic brain disorder and schizophrenia Psychogeriatrics 2009 9 2 73 76 19604329 10.1111/j.1479-8301.2009.00282.x

[B146] Barak Y Aizenberg D Mirecki I Mazeh D Achiron A Very late-onset schizophrenia-like psychosis: clinical and imaging characteristics in comparison with elderly patients with schizophrenia J Nerv Ment Dis 2002 190 11 733 736 12436012 10.1097/00005053-200211000-00002

[B147] Teeple RC Caplan JP Stern TA Visual hallucinations: differential diagnosis and treatment Prim Care Companion J Clin Psychiatry 2009 11 1 26 32 19333408 10.4088/pcc.08r00673 PMC2660156

[B148] Cornic F Consoli A Tanguy ML Bonnot O Perisse D Tordjman S Laurent C Cohen D Association of adolescent catatonia with increased mortality and morbidity: evidence from a prospective follow-up study Schizophr Res 2009 113 2–3 233 240 19443182 10.1016/j.schres.2009.04.021

[B149] Remschmidt H Theisen F Early-onset schizophrenia Neuropsychobiology 2012 66 1 63 69 22797279 10.1159/000338548

[B150] Walterfang M Bonnot O Mocellin R Velakoulis D The neuropsychiatry of inborn errors of metabolism J Inherit Metab Dis 2013 36 4 687 702 23700255 10.1007/s10545-013-9618-y

[B151] McEvoy JP The costs of schizophrenia J Clin Psychiatry 2007 68 Suppl 14 4 7 18284271

